# Prostate cancer cells and exosomes in acidic condition show increased carbonic anhydrase IX expression and activity

**DOI:** 10.1080/14756366.2018.1538980

**Published:** 2019-01-02

**Authors:** Mariantonia Logozzi, Clemente Capasso, Rossella Di Raimo, Sonia Del Prete, Davide Mizzoni, Mario Falchi, Claudiu T. Supuran, Stefano Fais

**Affiliations:** a Department of Oncology and Molecular Medicine, National Institute of Health, Rome, Italy;; b National Research Council, Institute of Biosciences and BioResources, Naples, Italy;; c National AIDS Center, National Institute of Health, Rome, Italy;; d NEUROFARBA Department, University of Florence, Section of Pharmaceutical Chemistry, Florence, Italy

**Keywords:** Tumour microenvironment, carbonic anhydrase IX, cancer, exosomes, LNCaP

## Abstract

Acidity and hypoxia are crucial phenotypes of tumour microenvironment both contributing to the selection of malignant cells under a micro evolutionistic pressure. During the tumour progression, nanovesicles, called exosomes and the metalloenzyme carbonic anhydrase IX (CA IX) affect the tumour growth and proliferation. Exosomes are released into the tumour microenvironment and spilt all over the body, while CA IX is a tumour-associated protein overexpressed in many different solid tumours. In the present study, to better understand the relationships between exosomes and CA IX, it has been used an *in vitro* cellular model of cells cultured in different pH conditions. The results showed that the acidic microenvironment induced upregulation of both expression and activity of CA IX in cancer cells and their exosomes, together with increasing the number of released exosomes. These data strongly support the importance of CA IX as a cancer biomarker and as a valuable target of new anticancer therapies.

## Introduction

In the last decade, the importance of exosomes has gained attention for their role in intercellular communication. They are cell-derived nanovesicles (40–180 nm) shuttling proteins, DNA, and RNA that could be transferred to recipient cells and modulate their protein expression^1^^,^^2^. Exosomes membrane is composed of a lipid bilayer enriched in cholesterol and sphingomyelin (typically lipid rafts components^3^), in ceramide (essential for the insertion of microRNAs into exosomes^4^) and Bis (monoacylglycero) phosphate (BMP) (an exosome-specific marker^5^). Exosomes are released from cells in physiological and pathological conditions, including cancer. It has been demonstrated that cancer cells produce more exosomes as compared to normal cells, and cancer exosomes differ from normal exosomes in molecular composition and function^6^. The tumour microenvironment is acidic, a common feature of virtually all cancers^7^. The extracellular acidity has a strong influence on the tumour microenvironment fitness, with a critical role in the progressive microevolution of cancers, including the induction of increased exosome release^7–10^. Tumour pH ranges *in vivo* from 6 to 6.8, with a mean of 6.5 and it is correlated to tumour malignancy^11–17^. In addition to low pH, tumour microenvironment is characterised by hypoxia and low nutrients supply, and a typical sign of malignancy that is the so-called aerobic glycolysis, also called Warburg Effect, i.e. sugar fermentation in hypoxic condition^7^^,^^18–21^. These cells produce ATP converting glucose in lactic acid, rather than metabolising it in mitochondria through oxidative phosphorylation^20^^,^^22–25^. However, this process implies an increase of glucose absorption to sustain energy requirement by tumour cells. The most important product of tumour metabolism is lactate leading in turn to H^+^ accumulation in the extracellular microenvironment with a direct consequence of the low pH^26^. High levels of carbonic dioxide produced during mitochondrial respiration of oxygenated cancer cells also contribute to a substantial release of H^+^ into the tumour microenvironment^27–32^. Low extracellular pH, lactic and carbonic acid production, uncontrolled growth, low blood and nutrient supply, contribute to generate a tumour microenvironment extremely toxic to normal cells. The toxic microenvironment progressively selects malignant cells, able to survive in this adverse condition thanks to up-regulation of expression and activity of several proton extrusion mechanisms, which release protons and lactate into extracellular microenvironment avoiding cytosol acidification^33^^,^^34^. In fact, an anti-acidic approach based on either Proton Pump Inhibitors (PPI)^9^^,^^35^ or buffers leads to acidification of cancer cell cytosol followed by quick and non-conventional cell death. Moreover, the anti-acidic treatment sensitises cancer cells to chemotherapeutics^12^^,^^15^^,^^16^, supporting the use of PPI as a new strategy against cancer^36–38^. Among proton flux regulator^21^ there are vacuolar H^+^-ATPases (V-ATPases), Na^+^/H^+^ exchanger (NHE), monocarboxylate transporters (MCTs), carbonic anhydrase IX (CA-IX)^10^^,^^33^^,^^34^, and Na^+^/HCO_3_ co-transporters (NBC)^39^. Interference (i.e. inhibition) with one or more of these proton pumps leads to a potent inhibition of cancer growth in a variety of *ex vivo* and *in vivo* models^40–42^. In this context, a pivotal role has been attributed to the CA IX, which is a metalloenzyme well studied in cancer. CA IX belongs to the α-CA genetic family among the seven CA-families known up to date. It is a membrane protein characterised by an extracellular proteoglycan domain, an extracellular catalytic domain, a transmembrane domain, and a short intracytosolic tail. Its primary function, like the other 14 human isoforms, is to catalyse the reaction of CO_2_ with H_2_O to produce H_2_CO_3_, which instantly dissociates to H^+^ and HCO_3_^−^. The promoter region of the gene (CA 9) encoding for CA IX contains a hypoxia-responsive element, with CA9 mRNA expression highly upregulated by hypoxia-inducible factor-1 (HIF1)^43^. Hypoxic tumours express high amounts of CA IX yielding an increase of the intracellular concentration of HCO_3_^−^ and extracellular acidification. The formed protons (H^+^) are secreted into the extracellular space through the pump/vacuolar-type ATPase and/or a Na^+^/H^+^ exchanger, while the HCO_3_^−^ is shuttled back to the cytosol mostly via a chloride/bicarbonate exchanger, although other ion exchangers may be involved as well (for both proteins more isoforms are known, some of which present predominantly in tumours)^44^. Therefore, CA IX activity is one of the main players responsible for the extracellular acidity of hypoxic tumours. One CA IX inhibitor (SLC-0111) actually progressed to Phase Ib clinical trials for the treatment of hypoxic, metastatic tumours^45^^,^^46^. This study hypothesised that some of the proton exchangers, extremely active in malignant cancer cells, could be expressed on exosomes and actively modulated by the acidic conditions. To this purpose, we explored the expression and activity of CA IX in cancer cells and cancer-released exosomes showing that CA IX expression and related activity are up-regulated under the acidic condition in both cells and exosomes. These results support a key role of CA IX in cancer progression and as both a cancer biomarker and a therapeutic target.

## Materials and methods

### Cell line

Human prostate carcinoma cell line (LNCaP) is derived from a metastatic site (left supraclavicular lymph node) of a 50-year-old Caucasian male (blood type B+) with confirmed diagnosis of metastatic prostate carcinoma (Istituto dei tumori di Milano). Tumour cells were negative for Mycoplasma contamination as routinely tested by PCR (Venor^®^GeM, Minerva Biolabs, Germany). The cells were maintained in RPMI culture medium supplemented with antibiotics and 10% foetal calf serum (FCS) (Invitrogen, Milan, Italy). Experiments were performed in buffered medium at pH 7.4 and in RPMI 1640 medium without sodium bicarbonate (pH 6.5) supplemented with antibiotics and 10% foetal calf serum (FCS) (Invitrogen, Milan, Italy). The acid cell culture medium (pH 6.5) was obtained by the addition of 1 M HCl solution. The pH was measured with a pH 123 Microprocessor pH Metre (Hanna Instruments, Milan, Italy).

### Confocal microscopy analysis

Cells were fixed 10 min in 4% paraformaldehyde, blocked 30 min in PBS with 1% BSA and labelled overnight with M75 antibody in blocking solution^47^. After washing with PBS +0.02% Tween20, cells were labelled with anti-mouse conjugated with AlexaFluor-488 and then with DAPI + ProLong (P36931, ThermoFisher Scientific).

Images were taken by a FV1000 confocal microscope (Olympus, Tokyo, Japan), using a (Olympus) planapo objective 60x oil A.N. 1.42. Excitation light was obtained by a Laser Dapi 408 nm for DAPI, an Argon Ion Laser (488 nm) for Alexa 488, DAPI emission was recorded from 415 to 485 nm, AlexaFluor-488 emission was recorded from 525 to 550 nm.

### Exosomes isolation from supernatant cell culture media

After 5 days of cell cultures, supernatants were collected, and exosomes isolated as described in Théry et al. and Kusuzaki et al^48^. Briefly, after centrifugation of cell at 300 *g* for 5 min, supernatants were centrifuged at 1200 *g* for 15 min followed by 12,000 *g* for 30 min. Supernatants were then filtered using a 0.22 μm filter (Millipore Corp., Bedford, MA) and centrifuged at 110,000 *g* for 1 h in a Sorvall WX Ultracentrifuge Series (Thermo Fisher Scientific) in order to pellet exosomes. After one wash in a large volume of phosphate-buffered saline (PBS), exosomes were resuspended in PBS (50 μl) for subsequent experimental analysis. To eliminate exosomes of FCS, the FCS was filtered with 0.45 and subsequently 0.22 μm filters (Millipore Corp., Bedford, MA) and then ultracentrifuged at 110,000 *g* before its addition to the culture media.

### Western blot analysis

Lysates were prepared in CHAPS buffer (10 mM Tris-HCl [pH 7.4], MgCl2 1 mM, EGTA 1 mM, CHAPS 0.5%, glycerol 10%, β-mercaptoethanol 5 mM, PMSF 1 mM) containing protease inhibitor cocktail. Cell lysates and exosomes were subjected to electrophoresis on SDS-polyacrylamide gels and transferred to nitrocellulose membranes (Protran Whatman, Dassel, Germany). After blocking in 5% dry milk in PBS1X, membranes were hybridised with primary antibodies: M75^47^, anti-Tsg101 (4A10, GeneTex, USA), anti-GAPDH (GA1R, Santa Cruz Biotechnology, USA) and anti-actin (C4, Santa Cruz Biotechnology, USA) monoclonal antibodies. After incubation with appropriate peroxidase-conjugated anti-IgG (Amersham Biosciences, Milan, Italy), membranes were revealed using the ECL Chemiluminescent Substrate, (ThermoFisher Scientific).

### Enzyme activity

LNCaP cells and their exosomes were obtained from cellular cultures grown at pH 7.4 and 6.5 as described above. Cell and exosome extracts were prepared at 4 °C using the lysis buffer containing 1% Triton X-100, 10 mM Tris-HCl (pH 7.4), MgCl2 1 mM, EGTA 1 mM, CHAPS 0.5%, glycerol 10%, β-mercaptoethanol 5 mM, and supplemented with a cocktail of protease inhibitors. Lysates were collected and clarified by centrifugation at 16,300×*g* for 15 min at 4 °C. Aliquots of cell or exosomes extracts containing 1 µg of total protein were used to determining the hydratase activity. The enzymatic assay was performed at 0 °C using CO_2_ as substrate following the pH variation due to the catalysed conversion of CO_2_ to bicarbonate. Bromothymol blue was used as the indicator of pH variation. The production of hydrogen ions during the CO_2_ hydration reaction lowers the pH of the solution until the colour transition point of the dye is reached. The time required for the colour change is inversely related to the quantity and activity of CAs present in the sample. Wilbur–Anderson units were calculated according to the following definition: One Wilbur–Anderson unit (WAU) of activity is defined as (T0 − T)/T, where T0 (uncatalysed reaction) and T (catalysed reaction) are recorded as the time (in seconds) required for the pH to drop from 8.3 to the transition point of the dye (pH 6.8) in a control buffer and in the presence of enzyme, respectively. Enzyme activity was expressed as WAU/µg of total protein. Protein concentration was determined using the Bio-Rad protein assay.

## Results

### CA IX expression is up-regulated in human prostate carcinoma cell line (LNCaP) cultured in acidic condition

**Figure 1.**
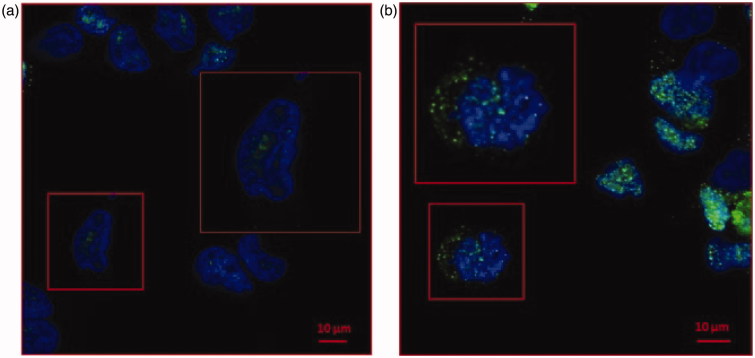
Confocal microscopy analysis of CA IX expression in LNCaP cell line cultured at pH 7.4 and pH 6.5. (a)LNCaP cells cultured at pH 7.4 showed low expression of CA IX (green signal) predominately nuclear (blue signal of DAPI). (b) CA IX expression in LNCaP pH 6.5 is higher compared to pH 7.4 and showed a strong cytoplasmic expression.

A human prostate cancer cell line (LNCaP) was cultured at two different pH conditions. LNCaP cells were buffered at pH 7.4 and 6.5, with no differences in terms of survival and viability as previously shown^6^. We checked the cell mortality (Trypan blue assay)(11) by cytofluorimeter analysis with again no differences in terms of mortality (1–2%) in both the cell pH culture conditions (data not shown). To evaluate the CA IX expression in LNCaP cells cultured at different pH, we first performed a Confocal Microscopy analysis. CA IX, predominantly expressed at the plasma membrane, can also be found into the cytoplasm and associated with the nuclear membrane. As shown in Figure 1, CA IX while expressed in both samples, independently from the culture condition, it was up-regulated in LNCaP cultured at pH 6.5 (Figure 1(b)). Remarkably, in LNCaP pH 6.5 (Figure 1(b)) CA IX showed an intracellular expression with a granular distribution as compared to the pH 7.4 culture condition (Figure 1(a)).

### CA IX expression is up-regulated in human prostate carcinoma cell line (LNCaP) cultured in acidic condition and in exosomes isolated from cell culture supernatant

**Figure 2.**
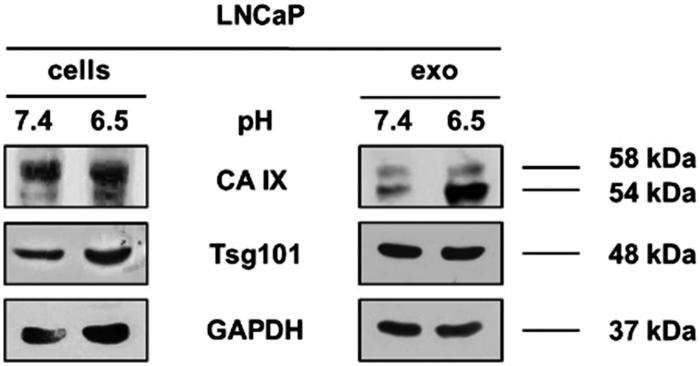
Western Blot of lysates from LNCaP pH 7.4 and LNCaP pH 6.5 cell and exosomes. CA IX expression was analysed in LNCaP lysates from cells and exosomes cultured at different condition. CA IX expression in increased both in cells and exosomes cultured at pH 6.5 compared to samples at pH 7.4. In particular, there is an increase of CA IX 54KDa band in samples at pH 6.5. Membranes were also incubated with anti-GAPDH, a housekeeping protein, and anti-Tsg101, a typical exosomal marker.

To validate the different CA IX expression, we performed a Western Blot Analysis; as shown in Figure 2, CA IX is characterised by the presence of two bands (58/54 kDa) with an up-regulated expression in cells cultured under acidic condition compared to pH 7.4 (1.5-fold higher calculated with densitometry software ImageJ) (data not shown). This result was consistent with previous reports showing that CA IX higher expression was associated with acidic microenvironment^49–51^.

Horie et al.^52^, demonstrated the presence of CA IX associated with exosomes fractions after OptiPrep density gradient centrifugation, so we analysed the whole exosomal purification lysates by Western Blot Analysis. Exosomes were characterised and quantified by Nanoparticle Tracking Analysis (NTA) (data not shown) and the expression of Tsg101, a typical exosomal marker. Consistent with the results obtained in cellular lysates, we showed that the CA IX expression in exosomes was up-regulated in acidic condition as compared to the 7.4 buffered condition (2.3-fold higher calculated with densitometry software) (data not shown). We also displayed that there was a different expression of the two CA IX bands (58/54 kDa) between cells and exosomes. In fact, cells extracts showed higher expression of 58 kDa CA IX while exosome preparation showed higher expression of the 54 kDa band. Interestingly, 54 kDa-form is always up-regulated in pH 6.5 condition both in cells and exosomes, suggesting to be the results of posttranscriptional changes of CA IX expression induced by the acidic state.

### CA enzymatic activity analysis of LNCaP cells and exosomes

**Figure 3.**
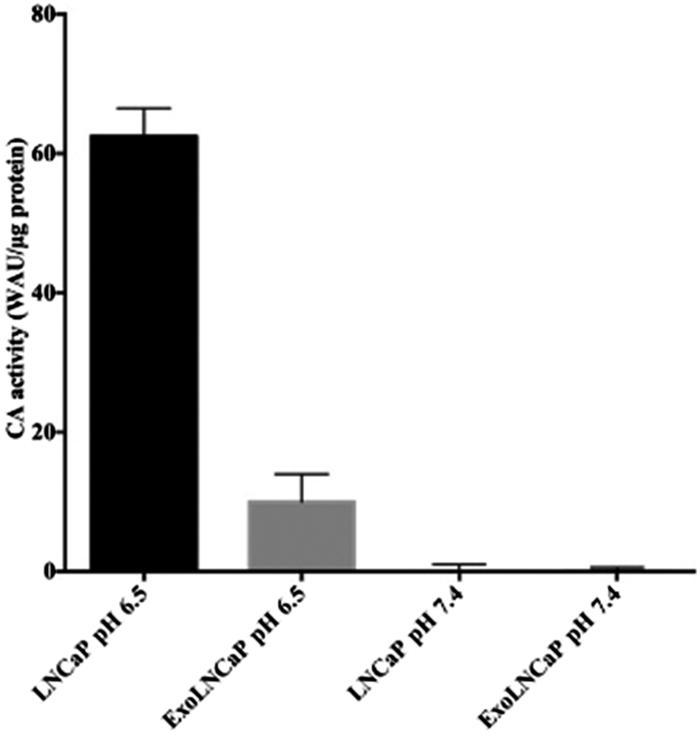
Analysis of CAIX enzymatic activity. CA IX activity is shown for LNCaP cells and their exosomes (ExoLNCaP) obtained from cellular cultures grown at pH 6.5 and 7.4. The results reflect a mean of three independent experiments.

As shown in Figure 3, the CA-activity has been found only in LNCaP cells and in the exosomes isolated from LNCaP cells grown at pH 6.5, while it was undetectable in LNCaP cells and in exosomes isolated from LNCaP cells grown at pH 7.4. These results are supported by the fact that the hypoxia condition, a state of all solid tumours, stimulates the hypoxia-inducible factor 1 (HIF-1), which induces the expression of the CA IX. The enzyme activity is responsible for the intracellular acidification and the acidity of the extracellular tumour microenvironment.

### Acidic microenvironment influences cellular and exosomal CA IX expression.

**Figure 4.**
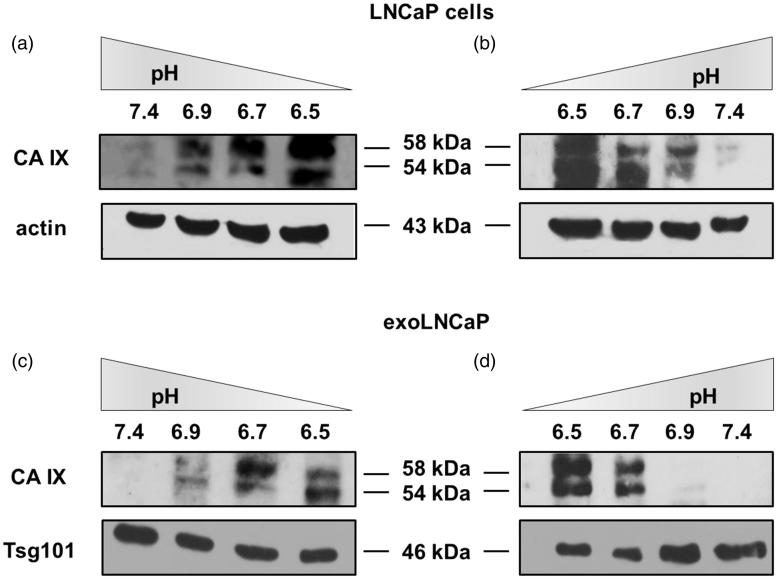
CA IX expression is related to microenvironmental pH. (a) Cells gradually cultured from pH 7.4 to 6.5 showed an increased CA IX expression. (b) CA IX decreased expression in LNCAP cells from pH 6.5 to 7.4. (c) Exosomes isolated from cells cultured from pH 7.4 to 6.5 showed an increased CA IX expression. (d) Exosomes isolated from cells cultured from pH 6.5 to 7.4 showed a decreased CA IX expression.

Considering that CA IX expression is related to microenvironmental pH, we cultured LNCaP cell line at different pH conditions^6^ ranging from 7.4 to 6.5 and vice-versa. After five days, cells were lysed to perform Western Blot Analysis and cell culture media were collected to obtain exosome purifications and perform WB analysis. CA IX expression in LNCaP cells progressively increased with the decrease of pH (Figure 4(a)), while decreasing with the increase of microenvironmental pH (Figure 4(b)). The same for the exosomes release at the different pH conditions from pH 7.4 to 6.5 (Figure 4(c)) and vice-versa (Figure 4(d)). NTA showed that pH-related change in CA IX expression was directly related to the number and size of exosomes released by the cells (i.e. higher number in acidic conditions lower at pH 7.4) (Supplementary Figure 1 and Figure 2, Supplementary Table 1 and Table 2).

## Discussion and conclusions

Tumour microenvironment (TME) exerts a key role in cancer pathogenesis, including initiation, progression, and response to therapy. As described in the literature, low pH, hypoxia and low blood and nutrients supply all contribute to setting a very hostile and selective microenvironment, that is common to virtually all cancers^11^. The adverse microenvironmental induces a Darwinian selection allowing the survival and proliferation of the cells most suited to live in those conditions. For example, malignant cells up-regulate the expression of many proton-efflux regulators to avoid the acidification of the cytosol, which leads to a quick cell death^33^^,^^53^. Among these proton-efflux regulators, there are several CAs, zinc metalloenzymes catalysing the conversion of carbon dioxide to bicarbonate and protons. CAs are expressed in almost all living organisms and they are involved in many physiological processes based on transport and pH balance (respiration, digestion, renal acidification, bone resorption etc^45^^,^^54^. Several isoforms are known and CA IX is markedly associated with solid tumours and by the way to the acidification of the tumour microenvironment pH^49–51^; thus representing a suitable target in cancer therapy^45^^,^^51^^,^^55–58^.

Recently, exosomes acquired a crucial role in the oncologic scenario since they are involved in cell-cell communication^59^, angiogenesis^52^, metastasis^60^ and drug resistance^61^. Moreover, exosomes contain a cell-type specific signature, thus representing a source of biomarkers in a variety of diseases, including cancer^2^^,^^6^^,^^62^^,^^63^. Furthermore, exosomes number and distribution could be included between the phenotypes common to virtually all cancers. We found, in fact, a higher number of exosomes in the plasma of cancer patients as compared to healthy donors^6^^,^^62^, correlating with the results obtained *in vitro* models^6^.

Since CA IX is a suitable marker of prostate cancer^64^ and its expression is up-regulated in hypoxia condition^64^^,^^65^, in the present paper we analysed how the acidic microenvironment of tumours could affect the CA IX expression and activity in human prostatic cancer cell line and exosomes released from them. The results showed different expression of CA IX in cells cultured in the acidic media (pH 6.5) respect to those at pH 7.4. The obtained results were consistent with the previous data showing that the low-pH is the most suitable condition for CA IX catalysis^49–51^. Moreover, the Confocal Analysis showed a remarkable expression of the protein in the cytoplasm of the cells cultured in the acidic microenvironment, with different internal distribution, suggesting that acidity influences CA IX expression and localisation within tumour cells. Notably, the CA IX expression in exosomes lysates reflected the CA IX expression at the cellular level in both acidic and physiological conditions. In addition, WB Analysis evidenced that the expression of the CA IX 54 kDa band increased in acidic condition at both cellular and exosome levels. All these experiments support the CA IX role in the *in vivo* tumour condition, since CA IX expression increased with progressive acidification and decreased with alkalinisation at both cellular and exosome levels. Thus, the tumour acidic microenvironment is the key factor in influencing the CA IX expression and activity in cancer cells and cancer-released exosomes. Previous reports have shown that CA IX is related to cancer development and progression^66^ and some malignant cancer activities such as cell spreading and migration^67^. Therefore, the possibility to obtain exosomes from plasma samples of tumour patients may offer the opportunity to consider CA IX a key tumour biomarker. Considering that CA IX is involved in the metastatic activity of cancer cells^52^^,^^68^, measuring its expression and activity in the exosomes may be helpful in monitoring cancer patients, as we have shown here for prostate cancer. Lastly, having exosomes a pivotal role in the metastatic process, both in preparing a metastatic niche and in inducing a tumour-like transformation in mesenchymal stem cells in target organs^60^, it is highly conceivable that the expression and activity of CA IX in tumour exosomes may be the key factor in the spreading of cancer all over the body. In conclusion, the future of anti-tumour strategies in primary cancers should be focussed in inhibiting the exosomes release and CA IX expression in malignant cancers. All strategies aimed at inhibiting microenvironmental acidity of tumours might represent a possible way to do it.

## Supplementary Material

Fais_Supplemental_Material.docx
