# Supplementary material for: Prostate cancer cells and exosomes in acidic condition show increased carbonic anhydrase IX expression and activity
Source: J Enzyme Inhib Med Chem. 2019 Jan 2;34(1):272–8. doi: 10.1080/14756366.2018.1538980 (PMC6327996; doi:10.1080/14756366.2018.1538980)
Supplement: Fais_Supplemental_Material.docx [file IENZ_A_1538980_SM9840.docx]

**Supplemental method**

***Nanoparticle tracking analysis***

Nanoparticle Tracking Analysis (NTA) from Malvern (NanoSight NS300) was used for size distribution and concentration measurements of nanovesicle samples in liquid suspension from the properties of both light scattering and Brownian motion. The NanoSight NS300 with a 405 nm laser instrument (Malvern Instruments, United Kingdom) was used to detect nanovesicles. Five videos of typically 60 s' duration were taken. Data was analysed by NTA 3.0 software (Malvern Instruments) which was optimized to first identify and then track each particle on a frame-by-frame basis.

**Supplemental Figure 1. Exosomes release from cells is influenced by microenvironmental pH**

After 5 days, supernatants from cell culture media were collected to isolate exosomes; then exosomes were quantified using NTA. Cells cultured from pH 7.4 to 6.5 showed a progressive increase of exosomes number (left panel), conversely going from pH 6.5 to 7.4 there was a reduction of exosomes release from cells (right panel).


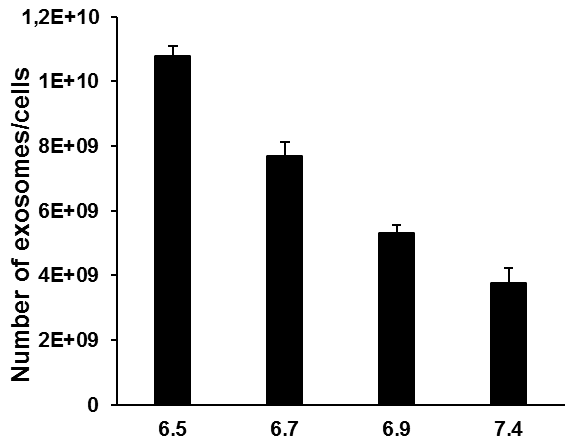


**pH**


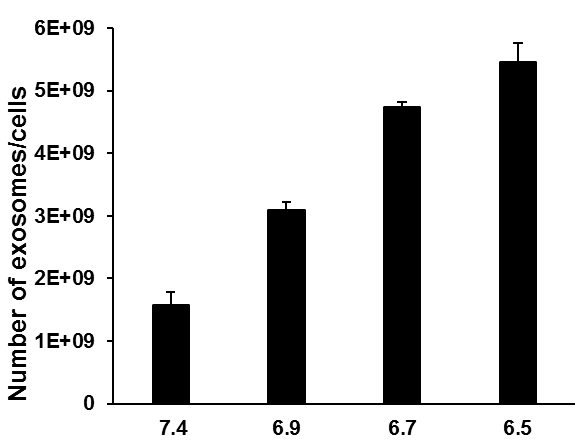


**pH**

**Supplementary Table 1. Increased release of exosomes from cells cultured from pH 7.4 to pH 6.5**

Summary of exosomes number quantified using NTA, from cells cultured from pH 7.4 to 6.5; there was a progressive increase of exosomes release from cells.

| **pH** | **Number of exosomes** |
| --- | --- |
| **7.4** | 1.58x10^9^±2.5x10^8^ |
| **6.9** | 3.10x10^9^±1.2x10^8^ |
| **6.7** | 4.74x10^9^±7.68x10^8^ |
| **6.5** | 5.45x10^9^±3.05x10^8^ |

**Supplementary Table 2. Progressive reduction of exosomes released from cells cultured from pH 6.5 to pH 7.4.**

Summary of exosomes number from cells cultured from pH 6.5 to 7.4. Exosomes were quantified using NTA and there was a progressive reduction of exosomes number going from pH 6.5 to 7.4.

| **pH** | **Number of exosomes** |
| --- | --- |
| **6.5** | 1.08x10^10^±3.33x10^8^ |
| **6.7** | 7.69x10^9^±4.31x10^8^ |
| **6.9** | 5.30x10^9^±2.55x10^8^ |
| **7.4** | 3.78x10^9^±4.67 x10^8^ |

**Supplemental Figure 2. Size of exosomes released from cells cultured at pH 7.4 and 6.5.**

Exosomes isolated from cells at pH 6.5 have a smaller size (97.1 ± 2.4 nm) compared to exosomes from cells at pH 7.4 (106.9 ± 3.1 nm).

**
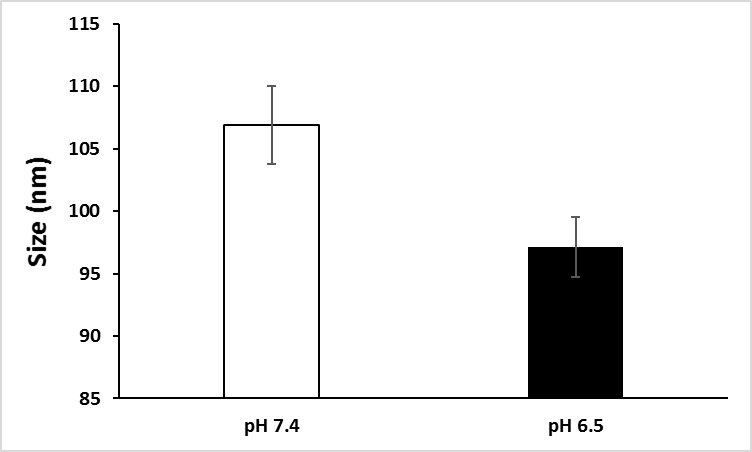
**

**Supplemental Figure 3. Densitometry analysis using ImageJ software.**
